# Multimodal perioperative management prevents antiendotoxin immunity exhaustion and systemic inflammation after major abdominal surgery

**DOI:** 10.1186/cc12910

**Published:** 2013-11-05

**Authors:** Pavlo Melnychenko, Alexander Potapov, Andrey Babanin

**Affiliations:** 1 Anesthesiology Department, Crimea State Medical University, Simferopol, Ukraine

## Background

One of the perspective directions for the improvement of surgical patients' treatment results is a multimodal approach in perioperative management including wide administration of regional anesthesia, early enteral feeding and modification of infusion therapy. The goal of this study is the assessment of the multimodal approach effect on antiendotoxin immunity and systemic inflammation after major abdominal surgery.

## Materials and methods

Open nonrandomized research. In the control group (*n *= 52), perioperative management was carried out with perioperative starvation, total intravenous anesthesia and analgesia on the basis of opiates. In the multimodal approach group (*n *= 40) we used a thoracic epidural analgesia in combination with early enteral feeding and preoperative infusion of HES 130/0.42 of 15 ml/kg body weight. In vein blood tests we analyzed C-reactive protein (CRP) and antibodies to lipopolysaccharide of *Escherichia coli *by IgM (anti-LPS-IgM) class. Data are submitted in the form of the median and 95% CI. The Mann-Whitney U criterion is used for comparisons between groups.

## Results

The median maintenance of CRP was 135.7 mkg/ml (95% CI = 153.5 to 249.5) in the control group for 3 days after operation but in the multimodal approach group was significantly lower - 89.0 mkg/ml (95% CI = 56.9 to 212.4; *P *< 0.05). The median anti-LPS-IgM level was 0.087 MU (95% CI, 0.084 to 0.226) in the control group in the same time but in the multimodal approach group was significantly higher - 0.181 MU (95% CI, 0.153 to 0.241; *P *< 0.001). The obtained data can mean that the expressed system inflammatory reaction has negative impact on the postoperative period. Reduced antiendotoxin immunity increases terms of hospitalization as an independent factor. This also increases the number of complications and lethality in surgery.

## Conclusions

The multimodal approach that includes thoracic epidural analgesia, early enteral feeding and preoperative infusion of HES 130/0.42 after volume abdominal operations prevents exhaustion of antiendotoxin immunity and system inflammatory reaction.
